# Trends and Regional Disparities in the Treatment of Echinococcosis in Kazakhstan: A Nationwide Retrospective Study (2017–2024)

**DOI:** 10.3390/healthcare14101304

**Published:** 2026-05-11

**Authors:** Bekdaulet Akimniyazova, Temur Yeshmuratov, Talgat Medetbekov, Axaule Serikbayeva, Nurgulim Akhmad, Asya Dyussembayeva, Saltanat Kenbayeva, Nadira Aitambayeva, Saule Nurakysh, Aigul Tazhiyeva

**Affiliations:** 1 Department of Pulmonology, Kazakhstan Medical University “KSPH”, Almaty 050060, Kazakhstan; bekus777@mail.ru; 2 Department of Surgery, Asfendiyarov Kazakh National Medical University, Almaty 050000, Kazakhstan; t.a.medetbekov@mail.ru; 3 Department of Surgery with a Course in Anesthesiology and Resuscitation, Kazakh-Russian Medical University, Almaty 050000, Kazakhstan; 4 Department of Pulmonology, Asfendiyarov Kazakh National Medical University, Almaty 050000, Kazakhstan; akss_1205@mail.ru; 5 Department of Normal Anatomy Named After S.R. Karynbaev, Asfendiyarov Kazakh National Medical University, Almaty 050000, Kazakhstan; akhmad.n@kaznmu.kz (N.A.); dyusembaeva.a@kaznmu.kz (A.D.); makhanbetova.s@kaznmu.kz (S.K.); 6 Department of Public Health and Social Sciences, Kazakhstan Medical University “KSPH”, Almaty 050060, Kazakhstan; aitambaeva.nadira@gmail.com; 7 Department of Health Politics and Management, Asfendiyarov Kazakh National Medical University, Almaty 050000, Kazakhstan; saulenurakysh@gmail.com; 8 Department of Science, Asfendiyarov Kazakh National Medical University, Almaty 050000, Kazakhstan

**Keywords:** echinococcosis, echinococcal granulosa infection, surgery

## Abstract

**Highlights:**

**What are the main findings?**

- A marked decline in surgically treated cases of echinococcosis was observed in Kazakhstan (2017–2024).
- Significant regional disparities were identified, with higher rates in southern endemic regions and major cities.

**What are the implications of the main findings?**

- The decline may reflect both improved control measures and potential underdiagnosis or reduced access to care.
- Regional inequalities highlight the need to improve early detection and access to surgical treatment.

**Abstract:**

**Background:** Echinococcosis, caused by the *Echinococcus granulosus*, primarily affects the liver and lungs and remains an important public health problem in endemic regions. Surgical treatment remains the main therapeutic approach. **Objectives**: We aimed to analyze nationwide trends and regional disparities in the treatment and surgical management of echinococcosis in Kazakhstan and to identify factors associated with emergency surgical care. **Methods**: A retrospective analysis of national healthcare records of treated echinococcosis cases in Kazakhstan from 2017 to 2024 was conducted. The dataset included information on age distribution, hospitalization characteristics, surgical interventions (elective and emergency), and regional patterns. Organ-specific stratification was available in the national database. Descriptive and comparative statistical analyses were performed to evaluate temporal and regional differences. **Results**: Between 2017 and 2024, the number of treated echinococcosis cases demonstrated a decreasing trend. Significant regional differences in surgical management were identified, with southern regions, particularly Turkistan, showing a higher proportion of emergency surgical interventions (*p* < 0.05). Forecasting analysis suggested a further decline in treated case numbers; however, widening 95% confidence intervals and the appearance of negative projected values in later years indicated substantial uncertainty and limitations of the predictive model. **Conclusions**: The decline in treated echinococcosis cases may reflect improvements in disease control, but may also be associated with underdiagnosis or reduced access to healthcare services. Marked regional disparities in emergency surgical care highlight the need to strengthen surveillance systems and improve equitable access to timely diagnosis and treatment.

## 1. Introduction

The 2025 World Health Statistics and monitoring of progress toward the Sustainable Development Goals (SDGs) indicate that neglected tropical diseases (NTDs) continue to pose a significant global health challenge, affecting 1.495 billion people worldwide in 2023 despite ongoing control efforts [1,2]. The epidemiological significance of echinococcosis arises from its severe clinical manifestations, which can result in disability and mortality, and from its complex transmission dynamics involving both animal reservoirs and human hosts [3]. Echinococcosis affects over 1 million individuals worldwide, with an estimated annual economic burden exceeding $3 billion [4].

The primary medically significant species are *Echinococcus granulosus* and *Echinococcus multilocularis*, which cause cystic and alveolar echinococcosis, respectively [5]. The liver is the most commonly affected organ. Pulmonary and multi-organ involvement are also frequently observed and can complicate both disease progression and treatment [6]. In advanced or complex cases, surgical intervention is an essential aspect of management, especially when conservative or pharmacological treatments prove inadequate [7].

The surgical management of echinococcosis is influenced by disease stage, timing of diagnosis, and availability of specialized healthcare services. Delayed diagnosis and restricted access to care increase the risk of complications necessitating urgent surgical intervention. Therefore, the ratio of emergency to elective surgical procedures can function as an indirect measure of healthcare system effectiveness, encompassing early detection, referral processes, and regional accessibility to surgical care [8,9]. Although the burden of echinococcosis is well recognized, evidence regarding nationwide trends in surgically treated cases and regional disparities in access to surgical care remains limited, especially in Central Asian countries. Analyzing these patterns is crucial for assessing healthcare system performance, identifying service delivery deficiencies, and improving resource allocation [10].

To date, no nationwide studies have systematically assessed temporal trends in surgically treated cases of echinococcosis or regional disparities in surgical care in Kazakhstan.

This study aimed to analyze nationwide trends in the number of surgically treated cases of echinococcosis and to assess regional disparities in access to surgical care in Kazakhstan, with a focus on factors associated with emergency surgical interventions.

## 2. Materials and Methods

This retrospective population-based study examined temporal and regional trends in treated echinococcosis in Kazakhstan between 2017 and 2024. Secondary data were obtained from the official national statistical collection “Health of the population of the Republic of Kazakhstan and the activities of healthcare organizations,” issued annually by the Committee on Statistics of the Republic of Kazakhstan between 2017 and 2024. (Astana, Kazakhstan, 2017–2024). The database included regional information on hospitalized patients treated for echinococcosis and the type of surgical care provided.

Cases were categorized as elective or emergency according to the mode of hospital admission recorded in the national database. Emergency cases included patients requiring urgent hospitalization and surgical intervention because of acute complications, such as cyst rupture, infection, or compression of vital structures. Elective cases referred to planned hospital admissions for scheduled surgical treatment.

All hospitalized echinococcosis cases recorded during the study period were eligible for inclusion. Records with incomplete information, duplicate entries, or unclear classification of admission type were excluded from the analysis. Because the national dataset was aggregated, organ-specific localization of echinococcosis was not available.

Descriptive statistics were used to summarize the data. Continuous variables are presented as means (M) and standard deviations (SD), whereas categorical variables are reported as frequencies and percentages. Temporal trends in the annual number of treated cases were assessed using linear trend analysis. Interregional differences were evaluated using chi-square tests for categorical variables and one-way ANOVA or the Kruskal–Wallis test, depending on data distribution and variance assumptions.

Given the aggregated structure of the national dataset, the presence of small regional counts, and missing observations in some years, the inferential statistical findings should be interpreted cautiously. Therefore, the analytical approach of the study was primarily descriptive and exploratory. All statistical analyses were performed using IBM SPSS Statistics version 25.0 (IBM Corp., Armonk, NY, USA). A *p*-value of <0.05 was considered statistically significant. Forecasting analysis was conducted using a time-series trend extrapolation approach in Microsoft Excel 2016. The forecasting model was used for illustrative purposes only, and its limitations, including the possibility of implausible projected values such as negative case counts, were acknowledged.

## 3. Results

**Figure 1:**
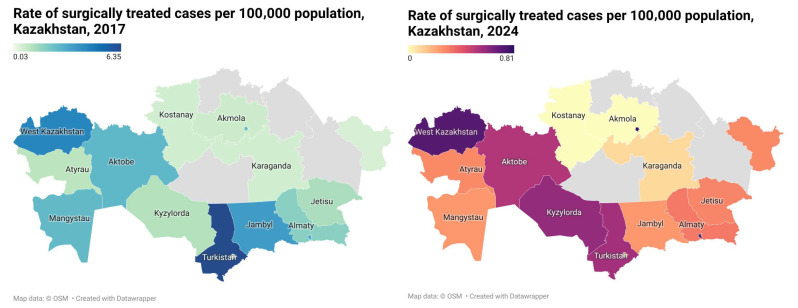
Regional comparison of surgically treated echinococcosis cases per 100,000 population in Kazakhstan, 2017 and 2024.

A comparative analysis of surgically treated echinococcosis cases in Kazakhstan (Figure 1) demonstrated a marked decline in case rates between 2017 and 2024. During this period, the national rate decreased from 2.29 per 100,000 population in 2017 to 0.37 per 100,000 in 2024, representing a 6.2-fold reduction. In 2017, the highest rates were observed in the southern and western regions of the country. Turkestan demonstrated the greatest regional burden, with 6.35 cases per 100,000 population and 126 treated cases. Elevated rates were also identified in West Kazakhstan (4.35 per 100,000), Almaty (3.60), and Zhambyl (3.58). Moderate rates were reported in Aktobe (2.70), Mangystau (2.61), and Astana (2.59), whereas substantially lower rates were observed in Karaganda (0.58), Kostanay (0.57), East Kazakhstan (0.50), and Abai (0.31). By 2024, most regions demonstrated a considerable reduction in surgically treated case rates. The highest rates were recorded in Astana (0.81 per 100,000 population) and Almaty (0.75), followed by West Kazakhstan (0.72) and Kyzylorda (0.59). In Turkestan, the rate declined from 6.35 to 0.56 per 100,000 population during the study period. Several regions, including Kostanay, reported very few or no surgically treated cases in 2024. Regions shown in grey in Figure 1 represent territories excluded from the dataset.

National data demonstrated an overall decline in the rate of surgically treated echinococcosis cases per 100,000 population between 2017 and 2023 (Table 1). However, temporal changes were not uniform and showed year-to-year variability across regions. In 2017, the highest rates were observed in Turkestan (6.35 per 100,000 population), West Kazakhstan (4.35), Almaty city (3.60), and Zhambyl (3.58). By 2023, these regions generally demonstrated lower reported rates, although the magnitude of change differed among regions and fluctuated during intermediate years. Regional dynamics remained heterogeneous throughout the study period. Several regions, including Aktobe, Mangystau, Zhetisu, and Kyzylorda, demonstrated variable trends characterized by both decreases and intermittent increases. Regions with initially lower rates, such as Abay, Akmola, Kostanay, and East Kazakhstan, maintained relatively low values across the study years, although interpretation in these areas is limited by small absolute case numbers. In major urban centers, including Almaty city and Astana city, rates also decreased over time but continued to demonstrate temporal variability. Overall, substantial interregional differences persisted during the study period, particularly because of consistently higher rates observed in southern regions such as Turkestan. A reduction in mean national values over time was identified, and overall trend analysis demonstrated statistical significance (*p* < 0.05). Nevertheless, these findings should be interpreted cautiously because of the aggregated structure of the dataset, variability in regional reporting, and incomplete observations for 2024. No consistent statistically significant trends were identified within individual regions (Table 1).

The number of treated echinococcosis cases in Kazakhstan decreased from 413 in 2017 to 284 in 2023 (Table 2). Although the overall trend was downward, temporal changes were characterized by year-to-year fluctuations, including a temporary increase in 2022 (330 cases). Moderate reductions were observed in 2018 (−41 cases) and 2019 (−30 cases), whereas a slight increase was recorded in 2022 (+6 cases). The most pronounced decrease was identified in 2024, when the number of reported cases declined to 74. However, this substantial reduction is likely associated with incomplete reporting and should therefore be interpreted cautiously. Growth and change rates further demonstrated temporal variability, with predominantly negative values throughout the study period. The only positive annual change was observed in 2022 (+1.85%), whereas the largest decline occurred in 2024 (−73.9%). Overall, the findings indicate a general downward tendency with intermittent fluctuations rather than a continuous uniform decline. Interpretation of these trends should take into account the aggregated nature of the national dataset and possible variability in regional reporting.

**Figure 2:**
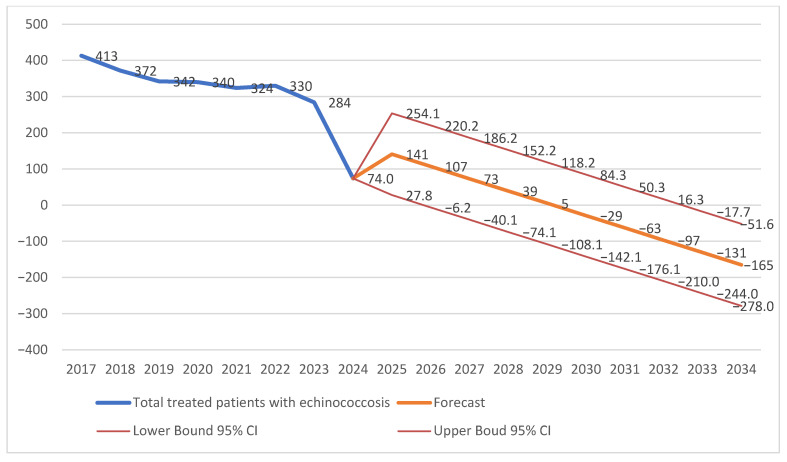
Trends in treated echinococcosis cases in Kazakhstan with a forecast to 2034.

The number of treated echinococcosis cases in Kazakhstan decreased from 413 in 2017 to 284 in 2023, although temporal changes were characterized by year-to-year fluctuations, including a temporary increase in 2022 (330 cases). In 2024, the number of reported cases declined sharply to 74; however, this value was likely influenced by incomplete reporting. Trend extrapolation analysis suggested a continued reduction in treated cases, with projected values of approximately 141 cases in 2025 and 107 cases in 2026. However, the forecasting model demonstrated substantial uncertainty, particularly in long-term projections that generated implausible negative values. Therefore, the forecast findings should be considered illustrative rather than predictive (Figure 2).

The number of elective surgical procedures for echinococcosis varied considerably across regions and study years (Table 3). In 2017, the highest numbers of elective operations were recorded in Almaty city (61 cases), Turkestan (47 cases), Zhambyl (30 cases), and Astana city (25 cases). By 2024, most regions had reduced elective surgical procedures. For example, the number of operations decreased from 61 to 16 in Almaty city, from 47 to 4 in Turkistan, and from 30 to 2 in Zhambyl. However, the marked decline observed in 2024 should be interpreted cautiously, as the data for that year may be incomplete. Regional trends were heterogeneous throughout the study period. Between 2017 and 2019, the number of elective procedures remained relatively stable with moderate fluctuations. Several regions demonstrated decreases during 2020–2021, followed by partial increases in 2022–2023. For instance, the Akmola region recorded 6 cases in 2023. Overall, larger urban centers and southern regions consistently reported higher numbers of elective procedures, whereas northern and less populated regions maintained lower case counts throughout the study period.

Analysis of the available data demonstrated substantial interregional variability in the number of emergency surgical procedures for echinococcosis (Table 4). The highest mean numbers of operations were recorded in Turkistan (Mean = 56.5) and Almaty region (Mean = 13), whereas the lowest values were observed in Kostanay, Astana city, East Kazakhstan, and Atyrau (1–2 operations). These findings indicate an uneven distribution of emergency surgical care across regions, potentially reflecting differences in healthcare accessibility and resource allocation. Higher standard deviation (SD) values were identified in Turkestan (SD = 6.36), Zhetisu (SD = 2.97), and the Almaty region (SD = 4.24), suggesting greater variability in reported cases. In contrast, Atyrau and East Kazakhstan demonstrated consistently low reported values across the available observations. Data for the period 2019–2021 were unavailable for most regions, resulting in an incomplete time series and limiting the assessment of temporal dynamics. Therefore, the analysis was primarily descriptive and based on the available annual observations. The absence of data during these years may be associated with disruptions in healthcare reporting or service provision during the COVID-19 pandemic. In urban centers such as Almaty, Astana, and Shymkent, the number of emergency procedures was lower than in major regional centers, particularly Turkestan and the Almaty region. This pattern may indicate a concentration of emergency surgical care within regional hospitals. Zhetisu demonstrated variability in reported cases across the available years (4, 6, and 2 operations), likely reflecting fluctuations in reporting practices or local differences in case detection. The apparent reduction in 2024 should also be interpreted cautiously because incomplete reporting may have affected the recorded values.

The age distribution of patients with echinococcosis between 2017 and 2024 is presented in Table 5. Most cases occurred among adults aged 18–59 years, accounting for approximately 54–63% of patients annually. Children aged 1–14 years represented the second-largest group (27–37%), whereas adolescents (15–17 years) and older adults (≥60 years) comprised smaller proportions. The mean age of patients increased from 27.8 years in 2017 to 34.4 years in 2024. However, interpreting the 2024 value requires caution because the total number of reported cases that year was substantially lower. From 2017 to 2023, the mean age remained relatively stable, ranging from 28 to 30 years, with an overall mean of 29.0 ± 1.2 years. The proportion of patients aged ≥ 60 years remained consistently low across the study period, although minor annual variations were observed. The age-distribution analysis was based only on cases with available demographic information; therefore, the total number of patients included in Table 5 was slightly lower than the number of treated cases reported in Table 2.

## 4. Discussion

A nationwide retrospective analysis demonstrated a decline in the rate of surgically treated echinococcosis cases in Kazakhstan between 2017 and 2024, accompanied by pronounced regional disparities in both case distribution and the proportion of emergency versus elective surgical interventions. This observed reduction should be interpreted with caution, as it may reflect not only potential improvements in disease control and early detection but also variations in reporting completeness, diagnostic coverage, and access to surgical care, particularly in recent years.

A key finding of this study is the substantial regional heterogeneity in surgical care. Regions such as Turkistan and Shymkent had a higher proportion of emergency surgical interventions, suggesting delayed diagnosis and limited access to specialized care. In contrast, major urban centers such as Almaty and Astana showed a higher proportion of elective procedures, suggesting a more developed healthcare infrastructure and earlier disease detection.

The ratio of emergency to elective surgical interventions may serve as a proxy indicator of access to timely surgical care. Higher proportions of emergency procedures have been associated with delayed diagnosis and worse clinical outcomes [11,12].

These findings are consistent with previous studies indicating that delayed diagnosis and disease progression increase the likelihood of complications requiring urgent surgical intervention [13]. Furthermore, evidence from non-endemic settings suggests that structured referral systems and early diagnostic capacity are associated with higher rates of planned surgical management [14].

Surgical intervention remains an essential component of echinococcosis management, particularly in advanced or complicated cases [15,16]. At the same time, ongoing advances in operative techniques may contribute to improved treatment outcomes [17]. In addition, recent evidence suggests that treatment strategies should be individualized, with both surgical and minimally invasive approaches considered depending on disease characteristics and patient condition [18]. Regional disparities in surgical care also reflect differences in spatial access to healthcare services. Previous studies have demonstrated that geographic accessibility plays a critical role in determining the availability of emergency surgical interventions [19].

The regional differences observed in this study likely reflect variations in healthcare system organization, resource allocation, and accessibility of specialized care, highlighting persistent inequalities across regions [20]. Similar disparities have been reported in endemic areas, where disease burden and healthcare infrastructure significantly influence treatment patterns [21]. Regional evidence from Central Asia indicates that trends in cystic echinococcosis are influenced by demographic changes, healthcare access, and temporal factors, including age and cohort effects [22]. These findings support the interpretation that the observed patterns in surgically treated cases may reflect broader epidemiological and healthcare system dynamics.

Echinococcosis remains a significant clinical challenge, particularly in pediatric populations, due to the complexity of disease progression and organ involvement, which often require advanced management strategies [7].

Evidence from endemic regions indicates that cystic echinococcosis is frequently underreported due to diagnostic challenges and limitations in surveillance systems [23]. This suggests that reductions in reported or treated cases may not necessarily reflect a true decrease in disease burden.

Several limitations warrant consideration. First, the analysis utilized aggregated national-level data, which constrained the assessment of patient-specific factors such as disease stage, cyst localization, comorbidities, and treatment outcomes. Furthermore, the lack of organ-specific classification, such as pulmonary versus hepatic echinococcosis, limited the depth of clinical interpretation. Second, case classification depended on administrative reporting systems, which may be subject to coding errors, inconsistencies, or regional variation in data recording practices. Third, the dataset lacked detailed information on diagnostic pathways, delays in access to care, and socioeconomic determinants that could influence access to surgical treatment. Fourth, substantial missing data for 2019–2021 in most regions reduced the completeness of the time series and limited the ability to conduct robust temporal trend analyses. Additionally, the observed decrease in 2024 may result from incomplete reporting rather than an actual reduction in disease burden. These factors should be considered when interpreting the findings. Finally, the forecasting component of the study was exploratory and should be interpreted with caution, as it relied on trend extrapolation rather than validated predictive modeling. Despite these limitations, this study constitutes, to the best of our knowledge, the first nationwide assessment of temporal patterns and regional disparities in surgically treated cases of echinococcosis in Kazakhstan. The findings underscore the need to strengthen early detection strategies and enhance equitable access to specialized surgical care across regions.

Future research should integrate epidemiological data with detailed clinical characteristics and expand patient-level datasets to facilitate a more comprehensive understanding of factors associated with emergency surgical interventions.

## 5. Conclusions

This study demonstrated an overall decline in the rate of surgically treated echinococcosis cases in Kazakhstan between 2017 and 2024, accompanied by considerable regional variation in case distribution and surgical care patterns. Higher proportions of emergency surgical interventions in several regions may indicate delays in diagnosis and limited access to planned treatment. These findings emphasize the importance of strengthening early detection strategies, improving referral systems, and expanding equitable access to specialized surgical care throughout the country.

## Figures and Tables

**Table 1:** Total treated patients with echinococcosis per 100,000 population by region in Kazakhstan.

Regions	2017	2018	2019	2020	2021	2022	2023	2024
Abay	2 (0.31)	1 (0.16)	4 (0.62)	3 (0.47)	2 (0.31)	4 (0.65)	2 (0.33)	-
Akmola	5 (0.68)	6 (0.81)	4 (0.54)	3 (0.41)	6 (0.82)	5 (0.64)	7 (0.89)	-
Aktobe	23 (2.70)	18 (2.08)	18 (2.06)	17 (1.91)	9 (1.01)	16 (1.73)	15 (1.61)	5 (0.53)
Almaty	37 (1.84)	24 (1.18)	22 (1.07)	20 (0.97)	21 (1.01)	19 (1.27)	18 (1.19)	5 (0.32)
Atyrau	6 (0.98)	6 (0.96)	4 (0.63)	5 (0.77)	1 (0.15)	1 (0.15)	2 (0.29)	2 (0.28)
East Kazakhstan	7 (0.50)	5 (0.36)	5 (0.36)	8 (0.59)	10 (0.73)	6 (0.82)	3 (0.41)	2 (0.28)
Zhambyl	40 (3.58)	40 (3.57)	27 (2.39)	19 (1.67)	30 (2.63)	23 (1.89)	17 (1.39)	3 (0.25)
Zhetisu	9 (1.32)	18 (2.65)	14 (2.06)	20 (2.95)	8 (1.18)	10 (1.43)	14 (2.00)	2 (0.29)
West Kazakhstan	28 (4.35)	9 (1.39)	18 (2.75)	11 (1.67)	15 (2.27)	21 (3.06)	12 (1.74)	5 (0.72)
Karaganda	8 (0.58)	14 (1.01)	14 (1.02)	10 (0.73)	17 (1.24)	17 (1.50)	12 (1.06)	1 (0.09)
Kostanay	5 (0.57)	3 (0.34)	4 (0.46)	3 (0.35)	4 (0.46)	3 (0.36)	1 (0.12)	-
Kyzylorda	9 (1.16)	15 (1.90)	13 (1.63)	7 (0.87)	13 (1.60)	9 (1.09)	2 (0.24)	5 (0.59)
Mangystau	17 (2.61)	14 (2.09)	11 (1.60)	14 (1.97)	6 (0.83)	9 (1.19)	7 (0.90)	2 (0.25)
Turkistan	126 (6.35)	111 (5.60)	102 (5.10)	100 (4.93)	94 (4.60)	88 (4.18)	86 (4.04)	12 (0.56)
Shymkent	1 (0.03)	3 (0.31)	3 (0.29)	5 (0.47)	3 (0.28)	15 (1.27)	5 (0.41)	1 (0.08)
Almaty city	64 (3.60)	60 (3.28)	68 (3.61)	68 (3.49)	51 (2.58)	55 (2.26)	55 (2.51)	17 (0.75)
Astana city	26 (2.59)	25 (2.37)	11 (0.99)	27 (2.33)	34 (2.87)	29 (2.19)	26 (1.87)	12 (0.81)
Mean ± SD	24.29 ± 31.18	21.88 ± 27.44	20.12 ± 26.11	20.00 ± 25.75	19.06 ± 23.44	19.41 ± 21.87	16.71 ± 22.11	5.29 ± 4.91

*p*-value (trend, overall) < 0.05.

**Table 2:** Dynamics of treated patients with echinococcosis cases in 2017–2024.

Year	Total Treated Patients with Echinococcosis	Absolute Increase	Growth Rate (%)	Rate of Change (%)	Absolute Number Corresponding to 1% Growth	Visualization Index
2017	413					1000
2018	372	−41	90.1	−9.93	4.13	900.7
2019	342	−30	91.9	−8.06	3.72	828.1
2020	340	−2	99.4	−0.58	3.42	823.2
2021	324	−16	95.3	−4.71	3.40	784.5
2022	330	6	101.9	1.85	3.24	799.0
2023	284	−46	86.1	−13.9	3.30	687.7
2024	74	−210	26.1	−73.9	2.84	179.2

**Table 3:** Elective operations for echinococcosis.

Regions	2017	2018	2019	2020	2021	2022	2023	2024
Abay	2 (0.79)	1 (0.43)	3 (1.50)	1 (0.47)	2 (1.00)	2 (0.98)	2 (1.18)	-
Akmola	2 (0.79)	5 (2.16)	2 (1.00)	2 (0.94)	3 (1.50)	3 (1.46)	6 (3.55)	-
Aktobe	18 (7.09)	10 (4.31)	12 (6.00)	8 (3.76)	4 (2.00)	4 (1.95)	4 (2.37)	3 (6.52)
Almaty	17 (6.69)	14 (6.03)	10 (5.00)	8 (3.76)	7 (3.50)	8 (3.90)	6 (3.55)	1 (2.17)
Atyrau	2 (0.79)	4 (1.72)	4 (2.00)	1 (0.47)		1 (0.49)		
East Kazakhstan	5 (1.97)	2 (0.86)	1 (0.50)	5 (2.35)	4 (2.00)	2 (0.98)	1 (0.59)	1 (2.17)
Zhambyl	30 (11.8)	29 (12.5)	18 (9.00)	8 (3.76)	20 (10.0)	20 (9.76)	13 (7.69)	2 (4.35)
Zhetisu	4 (1.57)	6 (2.59)	9 (4.50)	11 (5.16)	4 (2.00)	6 (2.93)	8 (4.73)	
West Kazakhstan	17 (6.69)	4 (1.72)	13 (6.50)	7 (3.29)	8 (4.00)	15 (7.32)	8 (4.73)	3 (6.52)
Karaganda	5 (1.97)	9 (3.88)	11 (5.50)	8 (3.76)	14 (7.00)	16 (7.80)	9 (5.33)	1 (2.17)
Kostanay	3 (1.18)	1 (0.43)	1 (0.50)	1 (0.47)	2 (1.00)	1 (0.49)	1 (0.59)	
Kyzylorda	8 (3.15)	12 (5.17)	11 (5.50)	7 (3.29)	5 (2.50)	7 (3.41)	2 (1.18)	4 (8.70)
Mangystau	7 (2.76)	7 (3.02)	7 (3.50)	10 (4.69)	4 (2.00)	2 (0.98)	5 (2.96)	
Turkistan	47 (18.5)	51 (22.0)	30 (15.0)	47 (22.1)	44 (22.0)	34 (16.6)	29 (17.2)	4 (8.70)
Shymkent	1 (0.39)	0	1 (0.50)	1 (0.47)	1 (0.50)	6 (2.93)	3 (1.78)	1 (2.17)
Almaty city	61 (24.0)	53 (22.8)	58 (29.0)	63 (29.6)	45 (22.5)	51 (24.9)	48 (28.4)	16 (34.8)
Astana city	25 (9.84)	24 (10.3)	9 (4.50)	25 (11.7)	33 (16.5)	27 (13.2)	24 (14.2)	10 (21.7)
Mean ± SD	14.94 ± 17.25	13.65 ± 16.45	11.76 ± 13.99	12.53 ± 17.22	11.76 ± 14.82	12.06 ± 13.96	9.94 ± 12.63	2.71 ± 4.25

**Table 4:** Emergency operations for echinococcosis.

Regions	2017	2018	2019	2020	2021	2022	2023	2024	Mean ± SD
Abay			-	-	-	-	-	-	-
Akmola	3 (2.36)	1 (0.81)	-	-	-	-	-	-	2.0 ± 1.41
Aktobe	5 (3.94)	7 (5.69)	-	-	-	-	-	-	6.0 ± 1.41
Almaty	16 (12.6)	10 (8.13)	-	-	-	-	-	-	13 ± 4.24
Atyrau	2 (1.57)	2 (1.63)	-	-	-	-	-	-	2.0 ± 0.00
East Kazakhstan	2 (1.57)	2 (1.63)	-	-	-	-	-	-	2.0 ± 0.00
Zhambyl	8 (6.30)	10 (8.13)	-	-	-	-	-	-	9.0 ± 1.41
Zhetisu	5 (3.94)	10 (8.13)	-	-	-	4 (100.0)	6 (100.0)	2 (7.14)	5.4 ± 2.97
West Kazakhstan	10 (7.87)	5 (4.07)	-	-	-	-	-	-	7.5 ± 3.54
Karaganda	2 (1.57)	5 (4.07)	-	-	-	-	-	-	3.5 ± 2.12
Kostanay	1 (0.79)	2 (1.63)	-	-	-	-	-	-	1.5 ± 0.71
Kyzylorda	1 (0.79)	3 (2.44)	-	-	-	-	-	-	2.0 ± 1.41
Mangystau	7 (5.51)	5 (4.07)	-	-	-	-	-	-	6 ± 1.41
Turkestan	61 (48.0)	52 (42.3)	-	-	-	-	-	-	56.5 ± 6.36
Shymkent		2 (1.63)	-	-	-	-	-	-	-
Almaty city	3 (2.36)	6 (4.88)	-	-	-	-	-	1 (3.57)	3.33 ± 2.52
Astana city	1 (0.79)	1 (0.81)	-	-	-	-	-	2 (7.14)	1.33 ± 0.57

**Table 5:** Age distribution of patients with echinococcosis for 2017–2024.

Year	1–14 Years	15–17 Year	18–59 Years	60–69 Years	70 Years	Total	Mean Age (Years)
2017	142 (34.3)	24 (5.8)	229 (55.4)	12 (2.9)	6 (1.45)	413 (100.0)	27.8
2018	115 (36.8)	-	184 (58.9)	13 (4.16)	-	312 (100.0)	28.2
2019	113 (36.1)	-	186 (59.4)	14 (4.47)	-	313 (100.0)	28.5
2020	94 (31.9)	2 (0.68)	186 (63.2)	12 (4.08)	-	294 (100.0)	29.5
2021	92 (32.3)	4 (1.40)	174 (61.2)	14 (4.92)	-	284 (100.0)	29.4
2022	99 (33.6)	1 (0.34)	176 (59.8)	18 (6.12)	-	294 (100.0)	29.6
2023	88 (35.0)	1 (0.39)	148 (58.9)	14 (5.57)	-	251 (100.0)	29.0
2024	19 (27.1)	1 (1.42)	38 (54.2)	11 (15.7)	1 (1.42)	70 (100.0)	34.4
2017–2024	-	-	-	-	-	2.231	29.0 ± 1.2

## Data Availability

All data generated or analyzed during this study are included in this article. Further inquiries can be directed to the corresponding author. The data utilized in this study originate from publicly accessible sources. Population statistics for Kazakhstan were obtained from the statistical collection “Health of the population of the Republic of Kazakhstan and the activities of healthcare organizations,” Committee on Statistics, Astana, Kazakhstan, 2017–2024. Available online: https://prg.kz/document/?doc_id=34505302&pos=3;156 (accessed on 1 January 2022). All data sources are publicly available and may be accessed for verification and further analysis.

## Abbreviations

The following abbreviations are used in this manuscript:

AE	alveolar echinococcosis
CE	Cystic echinococcosis
MTD	Measures to combat neglected tropical diseases
NTDs	Neglected tropical diseases
